# The sphingosine 1-phosphate receptor 2 is shed in exosomes from breast cancer cells and is N-terminally processed to a short constitutively active form that promotes extracellular signal regulated kinase activation and DNA synthesis in fibroblasts

**DOI:** 10.18632/oncotarget.25658

**Published:** 2018-06-29

**Authors:** Ashref El Buri, David R. Adams, Douglas Smith, Rothwelle J. Tate, Margaret Mullin, Susan Pyne, Nigel J. Pyne

**Affiliations:** ^1^ Strathclyde Institute of Pharmacy and Biomedical Sciences, University of Strathclyde, Glasgow, Scotland, UK; ^2^ School of Engineering & Physical Sciences, Heriot-Watt University, Edinburgh, Scotland, UK; ^3^ Electron Microscopy Facility, School of Life Sciences, MVLS, Joseph Black Building, University of Glasgow, Glasgow, Scotland, UK

**Keywords:** sphingosine 1-phosphate, sphingosine 1-phosphate receptors, exosomes, fibroblasts, cancer

## Abstract

We demonstrate here that the G protein-coupled receptor (GPCR), sphingosine 1-phosphate receptor 2 (S1P_2_, Mr = 40 kDa) is shed in hsp70^+^ and CD63^+^ containing exosomes from MDA-MB-231 breast cancer cells. The receptor is taken up by fibroblasts, where it is N-terminally processed to a shorter form (Mr = 36 kDa) that appears to be constitutively active and able to stimulate the extracellular signal regulated kinase-1/2 (ERK-1/2) pathway and DNA synthesis. An N-terminally truncated construct of S1P_2_, which may correspond to the processed form of the receptor generated in fibroblasts, was found to be constitutively active when over-expressed in HEK293 cells. Analysis based on the available crystal structure of the homologous S1P_1_ receptor suggests that, in the inactive-state, the N-terminus of S1P_2_ may tension TM1 so as to maintain a compressive action on TM7. This in turn may stabilise a closed basal state interface between the intracellular ends of TM7 and TM6. Cleavage and removal of the S1P_2_ N-terminal peptide is postulated to facilitate relaxation of TM1 and accompanying separation of TM6 and TM7. The latter transition is one of the key elements of G protein engagement and is required to open the intracellular coupling interface beneath the GPCR helix bundle. Therefore, removal at the N-terminus of S1P_2_ is likely to enhance G protein coupling. These findings provide the first evidence that S1P_2_ is released from breast cancer cells in exosomes and is processed by fibroblasts to promote ERK signaling and proliferation of these cells.

## INTRODUCTION

The bioactive lipid, sphingosine 1-phosphate (S1P) is formed by the sphingosine kinase-catalysed phosphorylation of sphingosine. There are two isoforms of sphingosine kinase, termed SK1 and SK2, which are encoded by different genes and exhibit distinct sub-cellular localisation and biochemical properties and can regulate overlapping and non-overlapping signaling pathways in cancer cells [1]. In addition to its biosynthesis, the level of S1P is controlled by degradation reactions catalysed by S1P lyase, to produce (*E*)-2-hexadecenal and phosphoethanolamine, and by S1P phosphatase, which dephosphorylates S1P to regenerate sphingosine [1]. S1P can be released from cells *via* specific transporters in the plasma membrane and then bind to and stimulate a family of G protein-coupled receptors (GPCRs), the S1P receptors (S1P_1_-S1P_5_) on cells to induce biological responses [1].

S1P_2_ is coupled to G_i_, G_q_ and G_12/13_ and can regulate phospholipase C, Rho, Rho-dependent kinase and extracellular signal regulated kinase (ERK-1/2) pathways [2–4]. The receptor is localised to the plasma-membrane and is internalised in response to ligand stimulation [5, 6]. This involves β-arrestin-2 and G protein-coupled receptor kinase 2 (GRK-2)-dependent mechanisms. S1P binding to S1P_2_ also inhibits the phosphatidylinositol 3-kinase/Akt pathway *via* a Rho-dependent activation of phosphatase and tensin homolog (PTEN) to inhibit cell migration [7, 8]. S1P_2_ is also involved in regulating the hippo pathway [10] and activation of the transcription factors, YAP and TAZ [9, 10].

There is substantial evidence demonstrating that S1P plays a significant role in cancer, including regulating transformation, epithelial-mesenchymal transition, invasiveness, cancer cell survival, replicative immortality, tumour neovascularisation and metabolism [11]. Nevertheless, the role of S1P_2_ in cancer is controversial with evidence demonstrating that this receptor can both promote and inhibit tumorigenesis. For example, S1P_2_ inhibits the motility of cancer cells [12, 13], and high expression of S1P_2_ in the nucleus of tumours from ER^+^ breast cancer patients is associated with improved prognosis [14]. However, recent studies have shown that S1P formed by host SK1 and acting *via* S1P_2_ prevents induction of the metastasis suppressor, Brms1 (breast carcinoma metastasis suppressor 1), thereby promoting metastatic spread [15]. SK1 activation and localization to the plasma membrane, with subsequent activation of S1P_2_ by released S1P (‘inside-out’ signaling) also upregulates transferrin receptor 1 (TFR1) expression, which contributes to SK1-mediated oncogenesis [16]. Furthermore, SK1-derived S1P, acting on S1P_2_, inactivates PP2A and prevents dephosphorylation of the oncogenic fusion protein, Bcr-Abl, thereby increasing its stability in CML [17]. We have also demonstrated that the function of S1P_2_ can change dependent on its subcellular localisation [18]. We reported that the SK2 inhibitor, (*R*)-FTY720 methyl ether (ROMe) or siRNA knockdown of SK2 promoted the accumulation of S1P_2_ in the nucleus of MDA-MB-231 breast cancer cells and this was associated with growth arrest [18]. Additionally, the S1P_4_ antagonist, CYM50367 or siRNA knockdown of S1P_4_ promoted nuclear localisation of S1P_2_, indicating that S1P_2_ and S1P_4_ are closely linked functionally to prevent accumulation of S1P_2_ in the nucleus, and to thereby promote breast cancer cell growth [18]. Whether the effect of nuclear localised S1P_2_ on growth is a cancer-specific phenomenon requires further investigation.

Recently, SK1 was demonstrated to be released as a catalytically active enzyme in vesicles shed by human breast carcinoma 8701-BC cells [19]. We therefore considered the possibility that S1P receptors might also be shed from cancer cells in exosomes to enable cancer cell/fibroblast communication, critical for metastasis. Exosomes are cell-derived vesicles of ~ 100 nm diameter which are released from cells by fusion of multi-vesicular bodies (MVB) with the plasma membrane [20–22]. They are formed from intraluminal endosomal vesicles and contain proteins, miRNA, lipids and other constituents that can enable cellular communication once released. This indicates that endosomal recycling is a critical step in exosome formation. Exosomes are taken up by recipient cells by membrane fusion and/or utilization of the transferrin endocytic machinery.

We demonstrate herein, for the first time, that S1P_2_ is shed from breast cancer cells in exosomes and, when exposed to fibroblasts, is processed to a shorter form and taken up by these cells, whereupon it promotes activation of the ERK-1/2 pathway and stimulates proliferation of these cells. These findings suggest a novel functional signaling role for the processed short form of S1P_2_. Future studies are required to evaluate the role of this processed short form in regulating inter-cellular communication between cancer cells and fibroblasts and whether it can promote fibroblast transformation and enhance metastatic spread.

## RESULTS

### S1P_2_ is shed from MDA-MB-231 breast cancer cells

**Figure 1:**
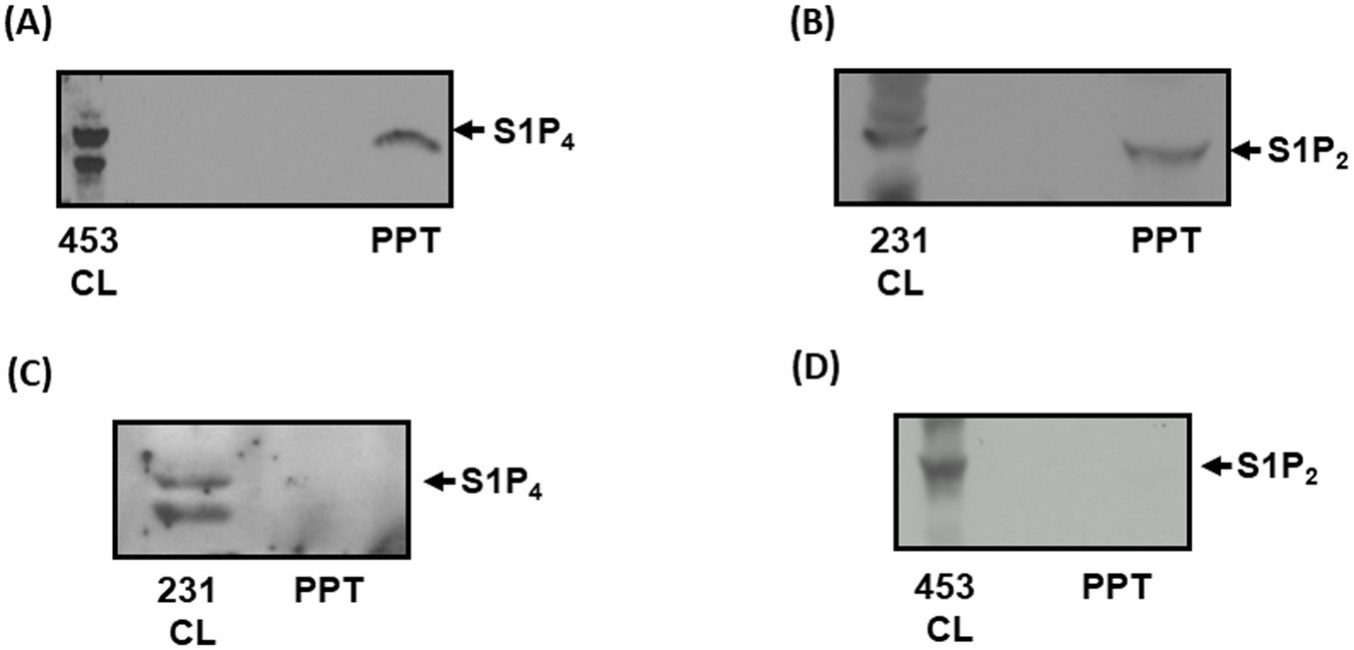
Identification of S1P_4_ and S1P_2_ shed from breast cancer cells **(A)** Western blot with anti-S1P_4_ antibody showing the presence of S1P_4_ in MDA-MB-453 cell lysate (453CL) and acid precipitates (PPT) of conditioned medium (CM) isolated after 48 h incubation with opti-MEM. **(B)** Western blot with anti-S1P_2_ antibody showing the presence of S1P_2_ in MDA-MB-231 cell lysate (231CL) and acid precipitates (PPT) of conditioned medium (CM) isolated after 48 h incubation with opti-MEM. **(C)** Western blot with anti-S1P_4_ antibody showing the absence of S1P_4_ in acid precipitates (PPT) of conditioned medium (CM) isolated from MDA-MB-231 cells after 48 h incubation with opti-MEM. The presence of S1P_4_ in cell lysates (231CL) of MDA-MB-231 cells is also shown. **(D)** Western blot with anti-S1P_2_ antibody showing the absence of S1P_2_ in acid precipitates (PPT) of conditioned medium (CM) isolated from MDA-MB-453 cells after 48 h incubation with opti-MEM. The presence of S1P_2_ in cell lysates (453CL) of MDA-MB-453 cells is also shown. Results are representative of 3 independent experiments.

Using proteomic analysis, Amorim et al. [23] found that extracellular vesicles [EVs, sedimentation at 100,000 g (100K) and 20,000 g (20K)] derived from HER2-expressing immortalized human mammary luminal epithelial HB4a and C5.2 cells contain S1P_3_ (exclusively in 20K EVs) and S1P_4_ (exclusively in 100K EVs) [23]. This was of interest because we have previously demonstrated that HER2 cooperates with S1P_4_ to regulate the ERK-1/2 pathway in MDA-MB-453 cells [24]. Indeed, we show here that S1P_4_ is released from MDA-MB-453 cells into conditioned medium (CM) (Figure 1A). S1P_4_ is expressed as 2 immunoreactive proteins with a Mr of 38-40 kDa in lysates of MDA-MB-453 cells and only one of these is released from these cells into CM (Figure 1A). Since, we have also demonstrated that S1P_2_ and S1P_4_ are functionally linked in MDA-MB-231 cells [18], we assessed whether these receptors are also released into CM from these cells. In this regard, S1P_2_ (Mr = 40 kDa) is released into CM from MDA-MB-231 cells (Figure 1B), while S1P_4_ is not released (Figure 1C). S1P_4_ is expressed as 2 immunoreactive proteins with a Mr of 38-40 kDa in lysates of MDA-MB-231 cells (Figure 1C). In addition, we found that S1P_2_ is not released into CM from MDA-MB-453 cells (Figure 1D). Therefore, these findings indicate that there is specificity in terms of the receptor sub-type released from these two breast cancer cell lines.

**Figure 2:**
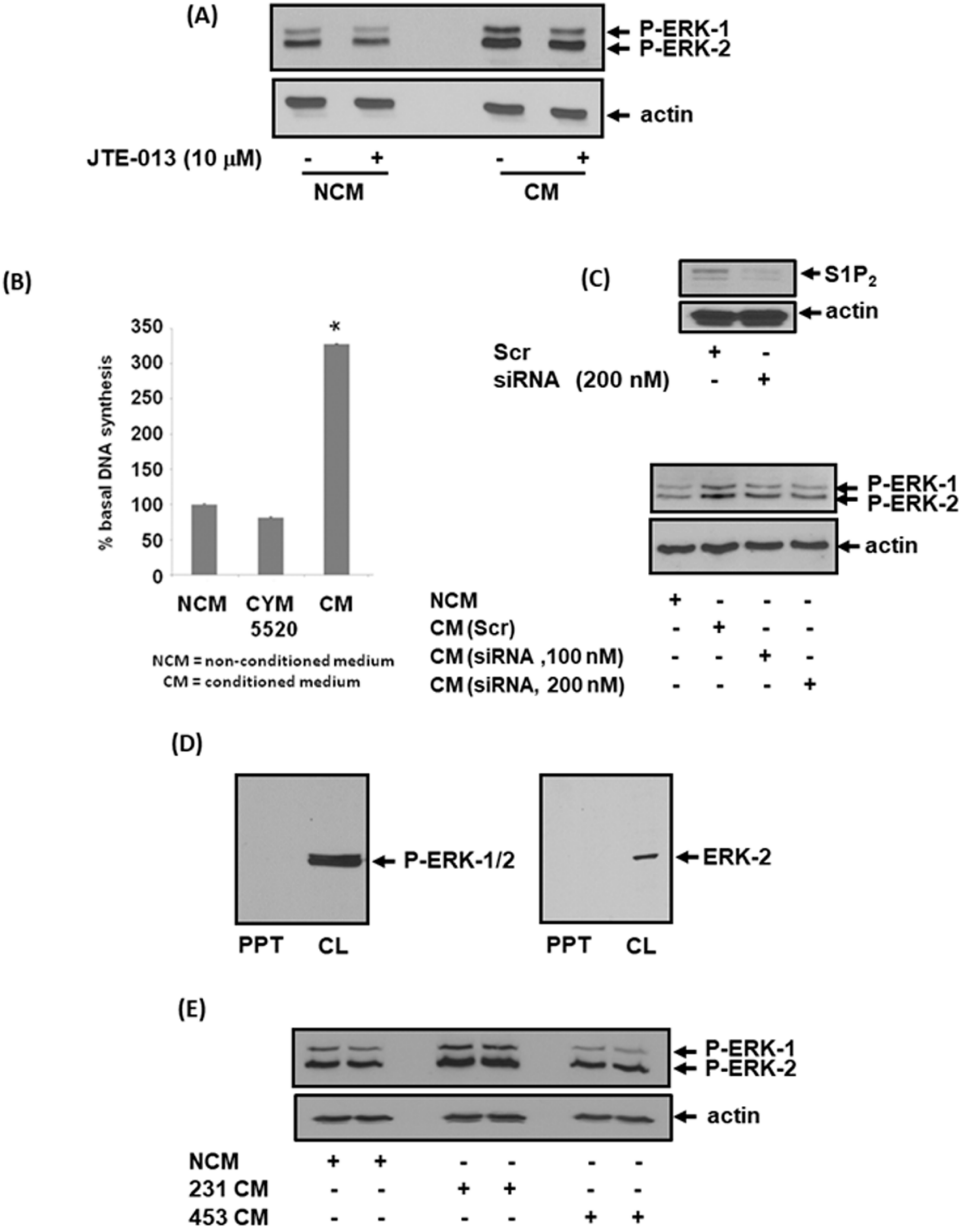
Effect of CM from MDA-MB-231 cells on ERK-1/2 activation and DNA synthesis in MEFs MEFs quiescent for 24 hr were treated with non-conditioned medium (NCM) or conditioned medium (CM) from MDA-MB-231 cells for 10 min. MEFs were pre-treated with and without JTE-013 (10 μM) for 15 min prior to addition of NCM or CM. MEFs were also treated with CYM5520 (10 μM) in the DNA synthesis experiments. CM was also isolated from MDA-MB-231 cells treated with scrambled or S1P_2_ siRNA for 24 h. **(A)** Western blot showing the effect of CM from MDA-MB-231 cells and JTE-013 on ERK-1/2 activation in MEFs. **(B)** Bar graph showing the effect of NCM, CM from MDA-MB-231 cells or CYM5520 on [^3^H]-thymidine incorporation into DNA synthesis in MEFs. Results are expressed as means +/- SEM for n=3. **(C)** Western blot showing the effect of CM on ERK-1/2 activation in MEFs. CM was isolated from MDA-MB-231 cells that had been treated with either scrambled siRNA (200 nM) or S1P_2_ siRNA (100 or 200 nM). Also shown is the effect of S1P_2_ siRNA on S1P_2_ expression levels in MDA-MB-231 cells. **(D)** Western blot showing that neither P-ERK-1/2 nor ERK-2 is released from MDA-MB-231 cells into CM as evidenced by their absence in the PPT. **(E)** Western blot showing the lack of effect of CM from MDA-MB-453 cells on ERK-1/2 activation in MEFs. Blots were probed with anti-phospho ERK-1/2 and anti-S1P_2_ antibodies. (A, C, D, E). Blots were re-probed with anti-actin antibody to ensure equal protein loading (A, C, E). Results (A, C, D, E) are representative of 3 independent experiments.

We next investigated the effect of CM isolated from MDA-MB-231 or MDA-MB-453 cells on mouse embryonic fibroblasts (MEFs) to establish whether S1P_2_ or S1P_4_ in CM might promote signaling in fibroblasts. In this regard, treatment of MEFs with CM from MDA-MB-231 cells induced a strong activation of ERK-1/2 (Figure 2A, Supplementary Figure 1) and promoted DNA synthesis (Figure 2B). Interestingly, the endogenous S1P_2_ receptor that is expressed in MEFs does not promote DNA synthesis in response to CYM5520 (Figure 2B), which is a potent and highly selective S1P_2_ allosteric agonist [25]. These findings suggest that the exosomal S1P_2_ receptor derived from breast cancer cells behaves differently from the endogenous S1P_2_ receptor that is expressed in MEFs.

We next assessed whether the S1P_2_ receptor released into CM from breast cancer cells requires S1P in order to activate ERK-1/2 signaling in MEFs. This was achieved using JTE-013, which is a competitive S1P_2/4_ antagonist [24]. The idea here is that if S1P is required, then activation of ERK-1/2 in MEFs in response to CM should be blocked by JTE-013. Basal ERK-1/2 signaling in MEFs was partially reduced by JTE-013 (Figure 2A, Supplementary Figure 1). However, JTE-013 had no effect on the ERK-1/2 activation induced by CM (Figure 2A, Supplementary Figure 1). These findings suggested either that stimulation of the ERK-1/2 pathway in fibroblasts was not mediated by the S1P_2_ shed from MDA-MB-231 cells into CM or that the released S1P_2_ might be processed to a form that does not require S1P and is therefore insensitive to inhibition by JTE-013. Confirmation that the S1P_2_ shed from MDA-MB-231 cells does in fact mediate the stimulatory effect of CM on ERK-1/2 activation in MEFs was obtained using siRNA to knockdown expression of S1P_2_ in MDA-MB-231 cells (Figure 2C, Supplementary Figure 2). Thus, CM isolated from siRNA S1P_2_ treated MDA-MB-231 cells failed to activate ERK-1/2 in MEFs (Figure 2C).

The increase in ERK-1/2 activation in MEFs in response to CM was not due to the uptake of phosphorylated ERK-1/2 as the kinase was not released from MDA-MB-231 cells into the CM, but was detected with either anti-ERK-2 or anti-phospho ERK-1/2 antibodies in lysates of MDA-MB-231 cells (Figure 2D). Specificity for S1P_2_ was confirmed by the demonstration that CM containing S1P_4_ from MDA-MB-453 cells failed to stimulate the ERK-1/2 pathway in MEFs (Figure 2E, Supplementary Figure 3). We therefore focussed our investigation on S1P_2_.

**Figure 3:**
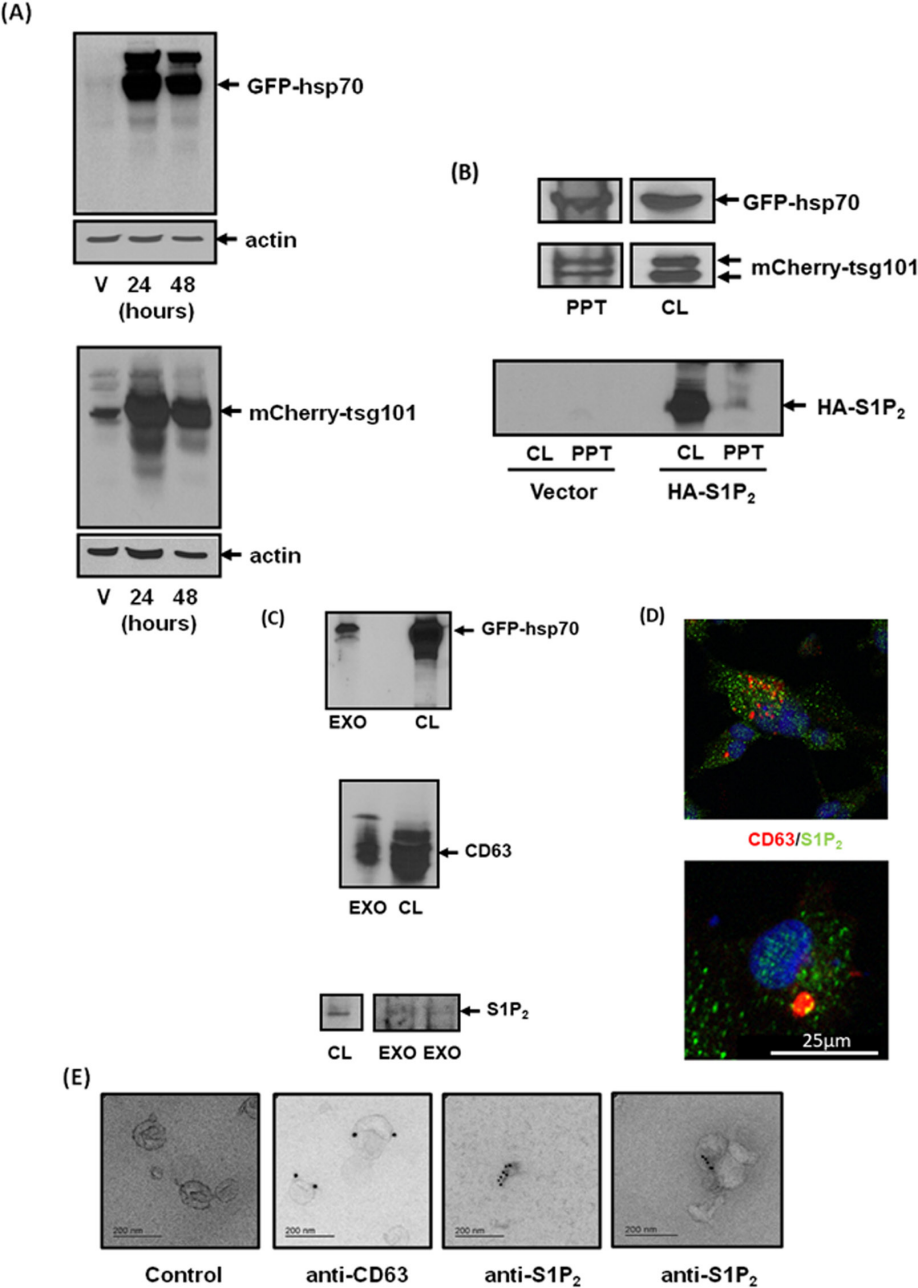
Identification of S1P_2_ in exosomes shed from MDA-MB-231 breast cancer cells **(A)** Western blot with anti-GFP or anti-mCherry antibodies showing the over-expression of GFP-hsp70 and mCherry-tgs101 in vector (V) or plasmid transfected MDA-MB-231 cells for 24 or 48 h. **(B)** Western blot with anti-GFP or anti-mCherry or anti-HA antibodies showing the presence of GFP-hsp70, mCherry-tsg101 or HA tagged S1P_2_ (Mr = 40 kDa) in transfected MDA-MB-231 cell lysate (CL) and acid precipitates (PPT) of CM. **(C)** Western blot with anti-hsp70, anti-CD63 and anti-S1P_2_ antibodies showing the presence of GFP-hsp70, CD63 or S1P_2_ in MDA-MB-231 cell lysates (CL) and isolated exosomes (EXO) from CM. **(D)** Immunofluorescence image of MDA-MB-231 cells stained with anti-CD63/TRITC (red) secondary and anti-S1P_2_/FITC (green) secondary antibodies showing co-localisation (yellow) of CD63 and S1P_2_ in large vesicles typical of MVBs. **(E)** Electron micrograph of immunogold staining with anti-CD63 [attached to secondary goat anti-mouse IgG-immune-gold particles (15 nm)] and anti-S1P_2_ antibodies [attached to secondary goat anti-rabbit IgG-immune-gold particles (10 nm)] in exosomes isolated from CM of MDA-MB-231 cells. Control represents exosomes that have not been incubated with primary antibody. Results are representative of 3 independent experiments.

To establish whether exosomes are released from MDA-MB-231 cells, we transiently over-expressed the exosomal markers, GFP-hsp70 or mCherry-tsg101 (Figure 3A). These proteins were subsequently found in CM isolated from these cells (Figure 3B), suggesting that export of S1P_2_ might indeed be *via* exosomes. Indeed, CM from MDA-MB-231 cells over-ovexpressing HA-tagged S1P_2_ also contained the receptor (Figure 3B). We next purified exosomes from the CM of MDA-MB-231 cells by ultracentrifugation. The exosome preparation contained S1P_2_ (Mr = 40 kDa) and the exosomal markers, CD63 and GFP-hsp70, which were detected by Western blot analysis (Figure 3C). MDA-MB-231 cells were also immunostained with anti-S1P_2_ antibody and anti-CD63 antibody (marker of MVB and exosomes) in order to track these proteins inside MDA-MB-231 cells. CD63 was present in large intracellular vesicles typical of MVBs that co-localised with S1P_2_ (Figure 3D). Finally, the exosome preparation was immunostained with anti-CD63 and anti-S1P_2_ antibodies using secondary gold linked antibodies and subjected to electron microscopy. Using this approach, purified exosomes were shown to contain CD63 and S1P_2_ (Figure 3E).

### Processing of exosomal S1P_2_ by fibroblasts

**Figure 4:**
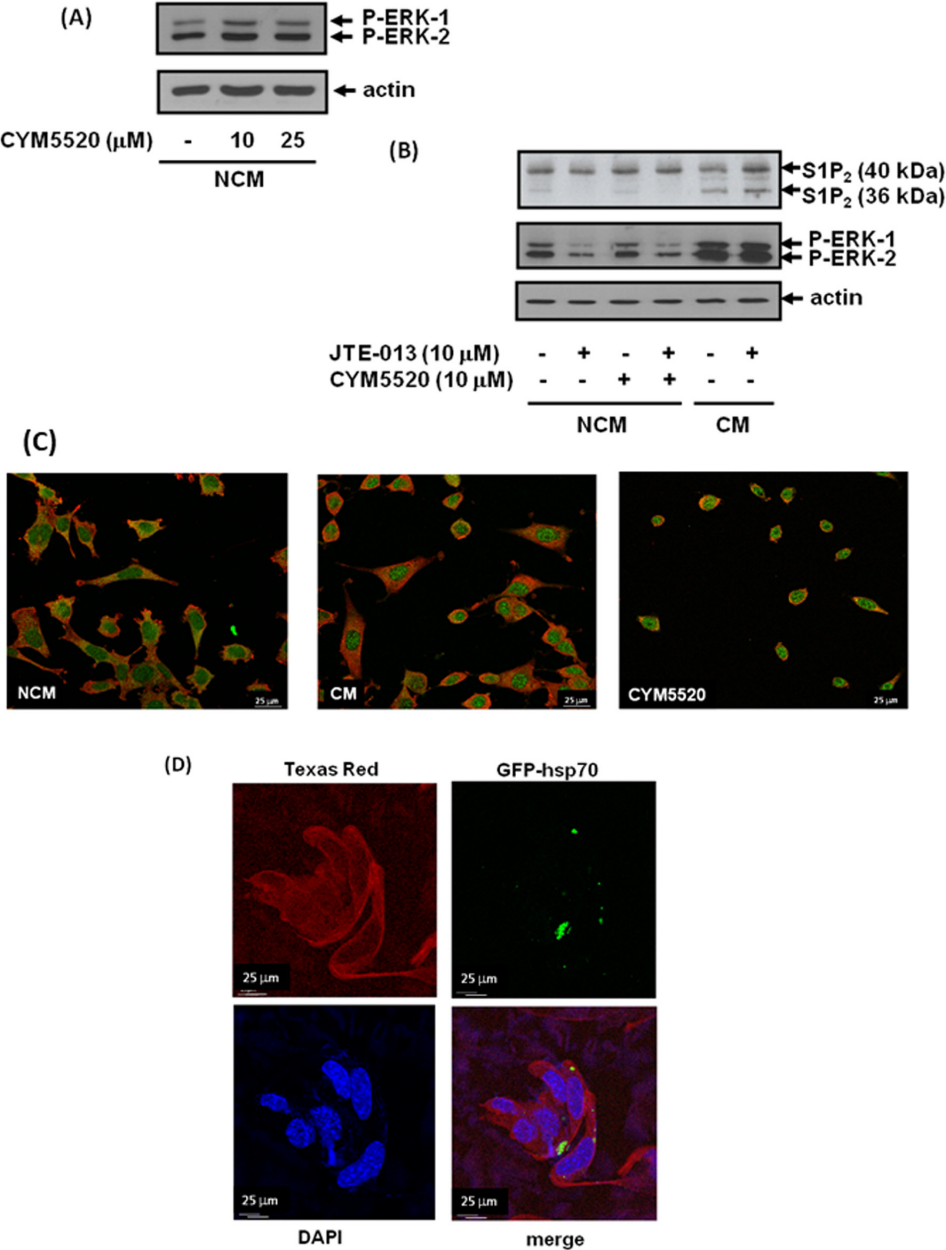
Uptake of exosomal S1P_2_ from breast cancer cells and processing by MEFs MEFs quiescent for 24 hr were treated with NCM or CM from MDA-MB-231 cells for 10 min. MEFs were pre-treated with and without JTE-013 (10 μM) or CYM5520 (10, 25 μM) for 15 min prior to addition of NCM or CM. **(A)** Western blot showing the lack of effect of CYM5520 alone on ERK-1/2 activation in MEFs. **(B)** Western blot showing the uptake into MEFs of the short S1P_2_ (Mr = 36 kDa) form and the activation of ERK-1/2 in response to CM isolated from MDA-MB-231 cells. (A) and (B) Blots were re-probed with anti-actin antibody to ensure equal protein loading. **(C)** Immunofluorescence images of control, CM-stimulated and CYM5520 (10 μM)-stimulated MEFs co-stained with anti-actin (TRITC secondary antibody, red) and anti-S1P_2_ (FITC secondary antibody, green) antibodies. **(D)** Fluorescence image of MEFs showing uptake of GFP-hsp70 by detection of GFP and co-stained with anti-actin antibody using a Texas Red conjugated secondary antibody or DAPI. Results are representative of 3 independent experiments.

MEFs express endogenous S1P_2_ (Mr = 40 kDa). However, the endogenous S1P_2_ receptor does not promote activation of the ERK-1/2 pathway in response to CYM5520 (Figure 4A). Therefore, the endogenous S1P_2_ receptor and exosomal S1P_2_ receptor released from MDA-MB-231 cells can be distinguished from one another by their different abilities to stimulate the ERK-1/2 pathway (Figure 2A) and DNA synthesis (Figure 2B) in MEFs.

We also treated MEFs with CM from MDA-MB-231 cells and performed western blot analysis with anti-S1P_2_ antibody in order to establish whether the breast cancer S1P_2_ receptor is taken up by fibroblasts from CM or exosomes. Indeed, S1P_2_ (Mr = 40 kDa) released from breast cancer cells was found to be taken up by MEFs, but appeared to be processed to a shorter form (Mr =36 kDa) (Figure 4B). The shorter form of S1P_2_ is likely to be proteolysed at the N-terminus of the receptor by proteinases expressed in MEFs, since the anti-S1P_2_ antibody is raised to the C-terminal region of S1P_2_.

Based on these findings, we propose that the short form of S1P_2_ is able to adopt a conformation that enables it to stimulate the ERK-1/2 pathway in MEFs. JTE-013 had no effect on the amount of the short S1P_2_ form taken up by the fibroblasts (Figure 4B). There is a low-level expression of the short form of endogenous S1P_2_ in MEFs and which is evident in cells treated with NCM (Figure 4B). Interestingly, the appearance of this shorter form is abolished when MEFs are treated with JTE-013 (Figure 4B). This is important for two reasons. First, the processing of endogenous S1P_2_ is distinguishable from the formation of the short form derived from breast cancer cells, which is insensitive to JTE-013. Second, the findings might suggest MEFs release S1P and that this stimulates a small fraction of S1P_2_, perhaps inducing a conformation and/or localisation that renders it susceptible to processing. In addition, JTE-013 reduces the basal activation state of ERK-1/2 in MEFs (Figure 2A, Figure 4B), suggesting that processing of the endogenous receptor renders it capable of stimulating the ERK-1/2 pathway. The allosteric agonist, CYM5520 (which is not a sphingophospholipid-mimetic ligand) did not activate the ERK-1/2 pathway (Figure 4A) and failed to increase formation of the endogenous short form of S1P_2_ (Figure 4B) suggesting that it does not induce a conformation of S1P_2_ that enables its processing. In contrast, the exosomal S1P_2_ receptor released from MDA-MB-231 cells is processed to the short form, presumably because it is immediately accessible to a putative protease expressed in MEFs and this is independent of S1P and therefore insensitive to JTE-013.

The endogenous S1P_2_ receptor in MEFs is functional, as CYM5520 induced rounding of MEFs and prevented actin localisation in lamellipodia, consistent with an inhibitory role of S1P_2_ in regulating a migratory phenotype in MEFs (Figure 4C). This might be mediated through G_12/13_ coupling and RhoA activation. CM from MDA-MB-231 cells also caused rounding of MEFs and prevented actin localisation in lamellipodia (Figure 4C), suggesting that the processed short S1P_2_ receptor form might exist in a conformation that is capable of concomitantly inducing both cell rounding and activation of the ERK-1/2 pathway. We also co-stained the fibroblasts with anti-S1P_2_ antibody and demonstrated that CM and CYM5520 promoted the redistribution of S1P_2_ into the nucleus of these cells (Figure 4C), although this is not linked with either ERK-1/2 signaling or DNA synthesis as CYM5520 does not stimulate these processes. The S1P_2_ receptor was detected in vesicle like structures, perhaps indicative of ‘on-going’ endocytosis. The significance of the re-localisation of S1P_2_ to the nucleus in response to CM in terms of promoting MEF proliferation requires further investigation.

To provide additional evidence that exosome cargo can be transferred from MDA-MB-231 cells to MEFs, we showed that exosomal GFP-hsp70 was also taken up by MEFs, detected using fluorescence microscopy (Figure 4D).

### Exosomal S1P_2_ from MDA-MB-231 cells activates fibroblasts

**Figure 5:**
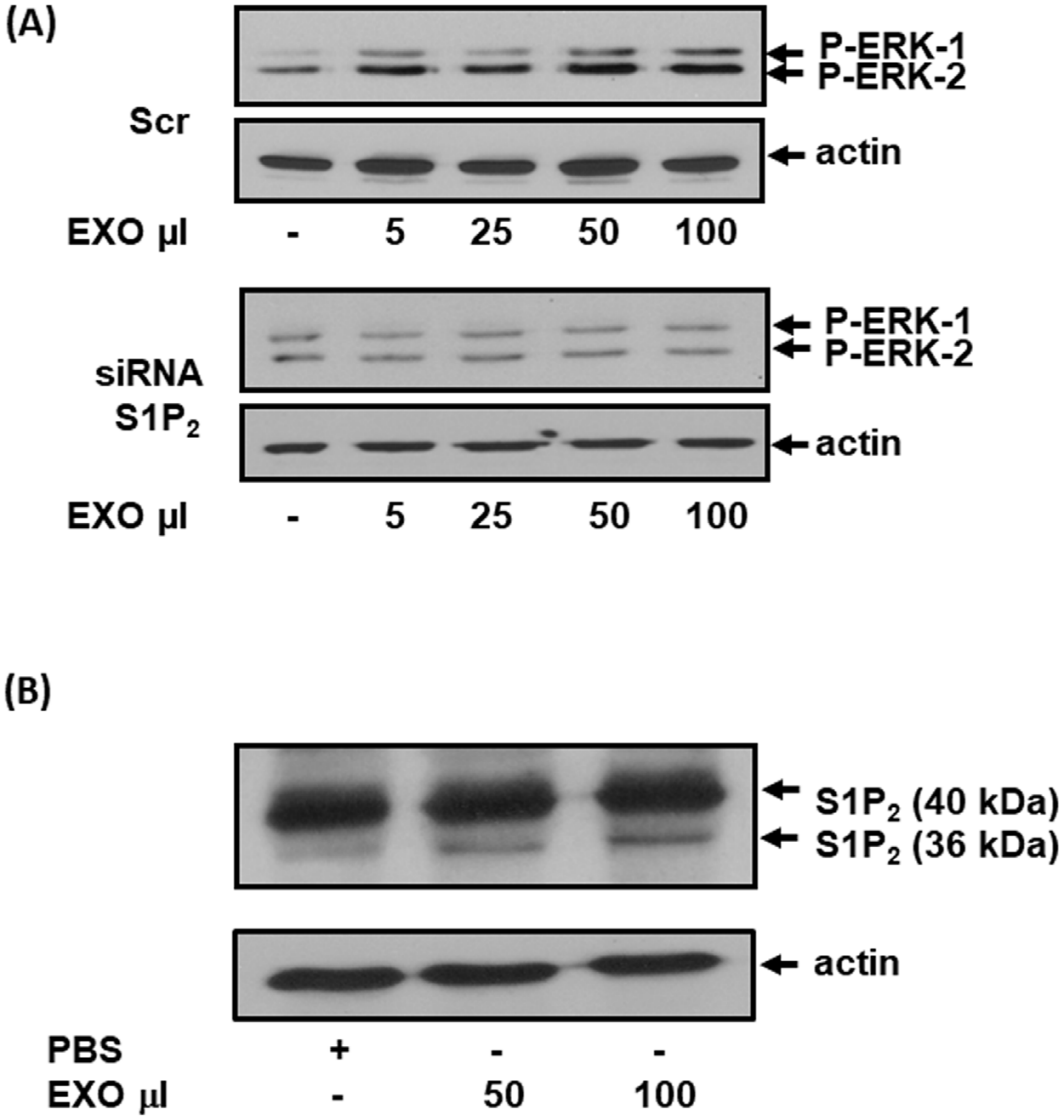
The effect of exosomal S1P_2_ on ERK-1/2 signaling and S1P_2_ processing in MEFs MEFs quiescent for 24 hr were treated for 15 min with exosomes isolated from CM of MDA-MB-231 cells that had been treated with either scrambled siRNA or S1P_2_ siRNA for 24 h. **(A)** Western blot showing the effect of exosomes (EXO, μl) from scrambled siRNA (100 nM) and S1P_2_ siRNA (200 nM)-treated MDA-MB-231 cells on ERK-1/2 activation in MEFs. Blots were probed with anti-phospho ERK-1/2 antibody. **(B)** Western blot showing the uptake into MEFs of the short S1P_2_ (Mr = 36 kDa) form from exosomes (EXO, 50 or 100 μl) isolated from MDA-MB-231 cells. Blots were probed with anti-S1P_2_ antibody. (A, B) Blots were re-probed with anti-actin antibody to ensure equal protein loading. Results are representative of 2-3 independent experiments.

We next tested the effect of purified exosomes on MEFs. In this regard, addition of purified exosomes from scrambled but not S1P_2_ siRNA-treated MDA-MB-231 cells promoted activation of ERK-1/2 in MEFs (Figure 5A, Supplementary Figure 4). We also subjected MEFs to western blot analysis after incubation with the purified exosomes. These experiments demonstrated that S1P_2_ was taken up by MEFs and processed to a short form, detected at Mr = 36 kDa, after incubation with exosomes (Figure 5B).

### Effect of truncated S1P_2_ on ERK-1/2 signaling in HEK293 cells

**Figure 6:**
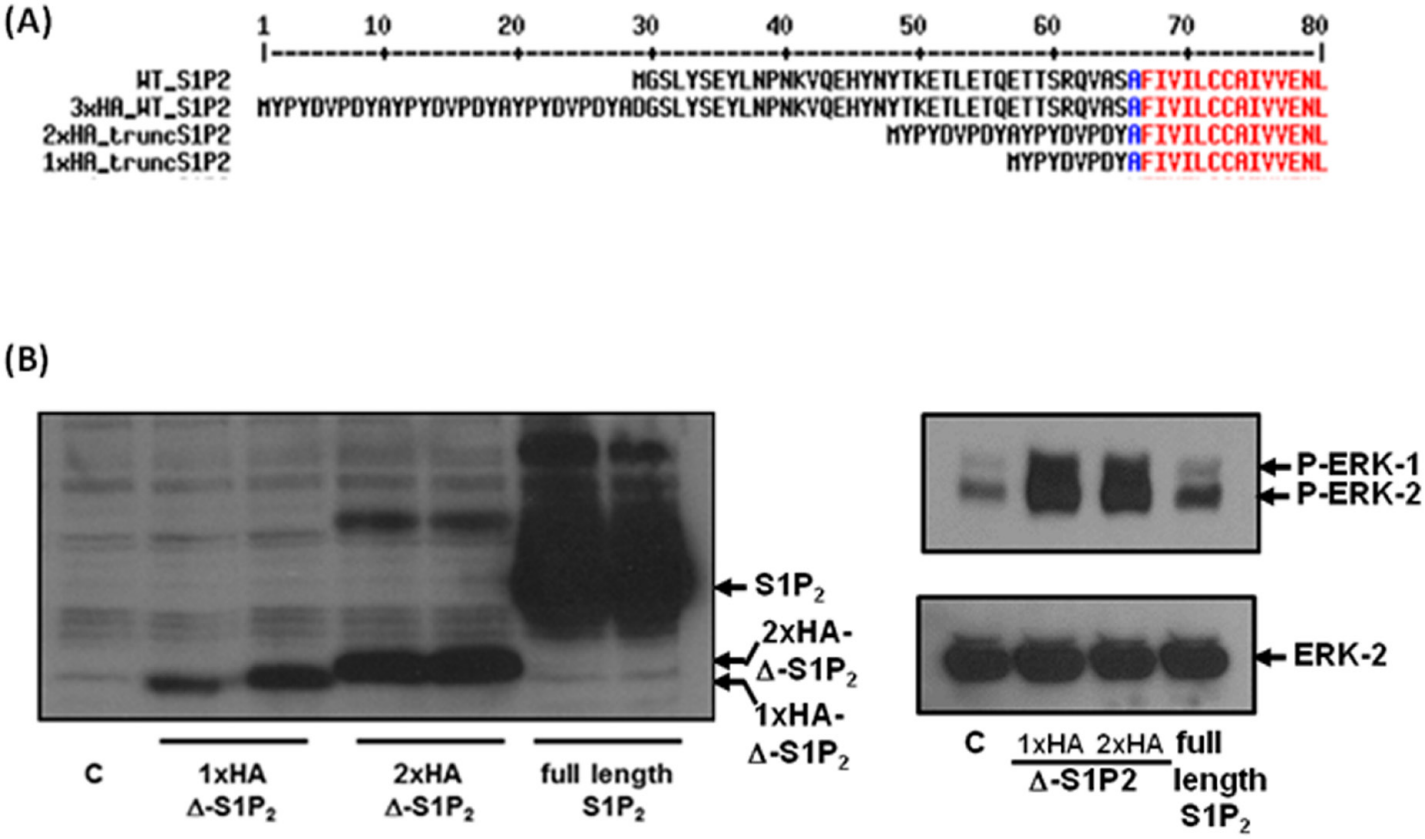
Overexpression of truncated S1P_2_ in HEK293 cells **(A)** Sequence alignment of the N-terminal region of WT and N-terminal HA-tagged full length and truncated S1P_2_ receptor constructs. **(B)** Effect of full length and truncated HA-tagged S1P_2_ on the activation of ERK-1/2 in HEK293 cells. Blots were probed with anti-HA-tag antibody, anti-phospho-ERK1/2 antibody and with anti-ERK-1/2 antibody to ensure equal protein loading. Results are representative of 3 independent experiments.

Inspection of the S1P_2_ receptor amino acid sequence reveals a potential intramembrane metalloprotease cleavage site corresponding to Ala-Ser-Ala-Phe-Iso-Val near the N-terminus (Figure 6A). We therefore sub-cloned a truncated S1P_2_ mutant corresponding to a processed form of the receptor at this site. Two truncated S1P_2_ forms, lacking the first 37 amino acids, were created with either one or with two N-terminal HA tags whereas the WT S1P_2_ has 3 N-terminal HA tags. Over-expression of the truncated 1x and 2x tagged HA-S1P_2_ receptor in HEK293 cells resulted in constitutive activation of ERK-1/2, while the WT S1P_2_ receptor failed to activate ERK-1/2 (Figure 6B).

### S1P receptor structure-function analysis

**Figure 7:**
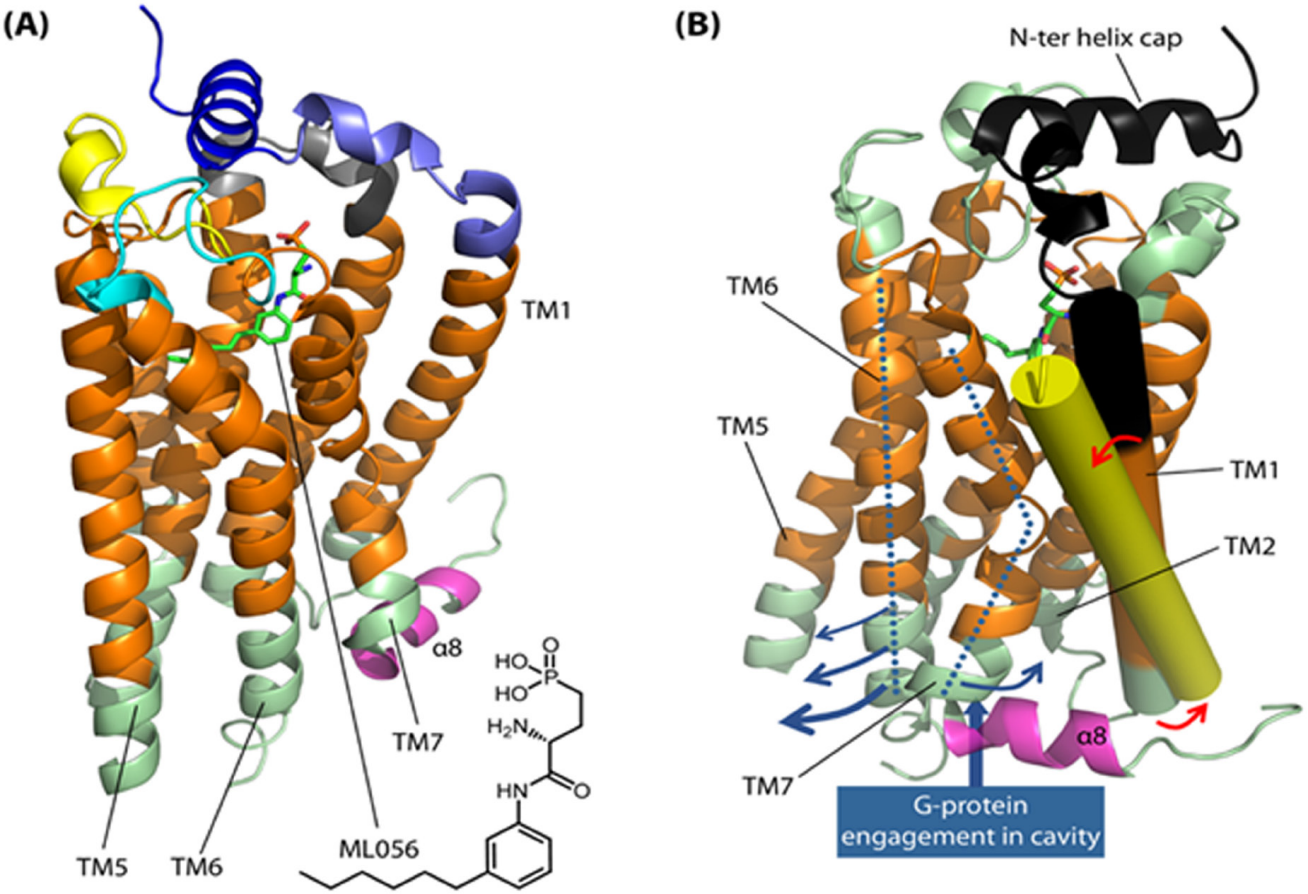
S1P_2_ structure-function analysis and constitutive activation hypothesis **(A)** The overall structural organisation for S1P_2_ is illustrated with respect to the co-crystal structure (PDB: 3V2Y) of the homologous S1P_1_ receptor with bound antagonist ML056 (green stick). Predicted membrane-spanning regions of the receptor are coloured orange. An N-terminal capping helix (S1P_1_ 22-YDIIVRHYNYT-32; dark blue ribbon) contributes key engagement residues for ligand phosph(on)ate head groups; residues involved in ligand head group binding from the core of the helix (S1P_1_ 28-HYNYT-32) are fully conserved in S1P_2_. The N-terminal capping helix is connected to TM1 by a short linker sequence (8 residues in S1P_1_, 9 residues in S1P_2_; light blue ribbon). Extracellular loops are coloured grey (ECL1), yellow (ECL2) and cyan (ECL3); ligand engagement features in these are strongly conserved between S1P_1_ and S1P_2_ and the overall fold, including two disulfide bridges in ECL2/3 (not shown), is also expected to be very similar in S1P_2_. We postulate that the N-terminal cap imposes a ‘spring clip’ positional constraint on TM1 that restrains movement in TM7 and helix-α8 (magenta ribbon) so as to stabilise a closed intracellular coupling interface and thus an inactive basal-state conformation; this involves close packing of TM7 against TM6 at the intracellular junction. **(B)** Rotated view of the S1P_1_ receptor illustrating the region (black ribbon) corresponding to the cleaved N-terminal segment in our constitutively active S1P_2_ receptor construct. ECL1-ECL3 are coloured light green in this panel; TM1 is highlighted (solid cylinder), with the N-terminal truncation site lying at the black/orange interface. Shown superimposed is the cognate TM1 (solid yellow cylinder) from the crystal structure (PDB: 3SN6) of the active-state β2 adrenoceptor bound to G protein. We postulate that N-terminal truncation permits relaxation of TM1 in S1P_2_ (red arrows), allowing adoption of an orientation akin to that for the active-state β2 adrenoceptor TM1. This in turn may allow relaxation of TM7 and separation of TM6 (blue arrows) to open the receptor's intracellular coupling interface.

The S1P_2_ receptor exhibits strong sequence homology to S1P_1_, which has recently been characterised by co-crystal structures (PDB: 3V2W, 3V2Y) obtained with the bound sphingophospholipid-mimetic antagonist, ML056 [26]. The typical 7TM GPCR architecture is conserved in the S1P_1_ crystal structure, but a distinctive feature is the presence of an N-terminal capping helix (S1P_1_ 22-YDIIVRHYNYT-32) that engages the phosph(on)ate head group of substrate and substrate-mimetic ligands with a hydrogen bond from Tyr29 (Figure 7A). A short 9-residue linker connects the N-terminal capping helix to the top of the TM1 helix. A closely related helix-capped extracellular organisation is seen in the lysophosphatidic acid receptor 1 structure [27]. Sequence alignment between S1P_2_ and S1P_1_ reveals that the core 28-HYNYT-32 residues of the N-terminal capping helix are fully conserved in S1P_2_ and that the structure of the interfacial extracellular loops (ECLs) that pack against this helix is also likely to be preserved, including two disulfide bridges that contribute to the fold of ECL2 and ECL3. Inspection of the S1P_1_ structure suggests that the N-terminal capping helix may tension TM1 and thence generate a compressive interface between the intracellular end of this helix and TM7 together with the angled intracellular helix-8. This may in turn stabilise an inactive basal-state packing of TM7 against TM6.

G protein engagement by GPCRs is known to involve docking to a cavity generated by movement in the TM helices, notably with an outward movement at the cytoplasmic end of TM5 and TM6 accompanied by movement of TM7 and the elbow with helix-8 in the opposite direction (towards TM1/TM2). Thus, comparison of inactive-state crystal structures for the β2 adrenoceptor with the receptor's structure when complexed to G protein (PDB: 3SN6) reveals a pronounced outward bend in TM6 away from TM7 [28]. The structure also suggests that the separation between TM6 and TM7 involves a concomitant displacement in TM7/helix-8 that is accommodated, in part, by tilting in TM1 together with outward bend at the cytoplasmic end of this helix (Figure 7B). In some GPCRs, an inactive basal-state is stabilised by inter-TM polar interactions (*e.g.* rhodopsin R^3.50^–E^6.30^); loosening of such ‘latches’ has been proposed to account for basal-state activity in the case of the β1 adrenoceptor [29]. For the S1P receptors, which lack inter-TM ionic latches, the N-terminal cap may conceivably serve as a ‘spring clip’ to tension TM1 and intracellular loop 1 against TM7/helix-8, thereby stabilising the closed TM7/TM6 interface. Proteolytic cleavage of the S1P_2_ N-terminal segment may allow relaxation in the position of TM1, with the resulting movement facilitating TM6/TM7 separation and thence opening of the intracellular coupling interface to confer constitutive activity (Figure 7B). In contrast, agonist-mediated S1P receptor activation is postulated to involve placement of hydrophobic ligand subunits into a cluster of hydrophobic and aromatic residues in the core of the helix bundle, including the TM6 residues, Trp269^6.48^ and Phe265^6.44^ (S1P_1_ numbering) [26]. These, residues are conserved in other class A GPCRs such as rhodopsin and the β2 adrenoceptor. In the case of the latter receptor, the crystal structure with bound G protein [28] reveals a significant displacement in the cognate Phe^6.44^ residue associated with the outward movement of TM6 and separation from TM7. Mutation of these residues in S1P_1_ decreases or abolishes ligand-induced ERK phosphorylation by the S1P_1_ receptor agonist, CYM5442, thereby highlighting the likely importance of TM6 movement to signaling. We therefore propose that whereas agonist binding may actively stabilise receptor conformational state(s) that engage partner signaling proteins, truncation of the S1P_2_ N-terminal sequence may perturb conformational equilibria by destabilising interfaces in receptor states(s) that preclude partner protein engagement.

The identity of the protease and precise site for proteolytic activation of S1P_2_ in fibroblasts have yet to be defined. We pre-treated cells with the ADAM inhibitor, GM6001 and phenoanthrolene which were ineffective in inhibiting CM-stimulated ERK-1/2 activation (data not shown). Nevertheless, the exploratory HA-tagged N-terminal S1P_2_ receptor, truncated at the site of a candidate metalloprotease consensus, was found to constitutively activate ERK-1/2. As shown in Figure 7B, however, the truncation site that we explored is actually predicted to lie within the membrane embedded region of TM1, so that cleavage at this specific site would likely require an intramembrane protease. Helix-destabilising features that might render the site more accessible to protease action appear to be lacking in the S1P_2_ TM1 sequence. It is possible, therefore, that the actual site of proteolysis may lie somewhat N-terminal to our exploratory site, potentially within the extracellular linking sequence that connects the N-terminal capping helix to the top of TM1.

## DISCUSSION

The major finding of this study is that S1P_2_ (Mr = 40 kDa) is shed from MDA-MB-231 cells and taken up by fibroblasts, where it appears to be processed at the N-terminus to a smaller constitutively active form (Mr =36 kDa). S1P_2_ is present in exosomes derived from MDA-MB-231 cells based on several lines of evidence. First, S1P_2_ and CD63 (a marker for exosomes) were co-localised in large vesicles in MDA-MB-231 cells that are typical of MVBs. Second, EM analysis of exosome preparations demonstrate vesicle-like structures that contain S1P_2_ and CD63, and this is supported by evidence showing S1P_2_, Hsp70 and CD63 are detected in CM and the exosome preparation by Western blot analysis with specific antibodies. Topographical arrangement of S1P_2_ in exosomes and uptake into the plasma-membrane of recipient cells would place its C-terminal tail on the cytoplasmic side of the recipient cell, thereby enabling the short form of S1P_2_ to initiate signaling into the intracellular compartment of MEFs.

The finding that S1P_2_ can be released from breast cancer cells in exosomes raises questions as to what factors determine this release. In this regard, Kajimoto et al. [30] recently demonstrated that continuous activation of the S1P_1_ receptor and subsequent G protein signaling by Gβγ subunits/Rho family GTPases regulates F-actin formation on multi-vesicular bodies required for cargo sorting into exosomal intralumenal vesicles. Continuous G protein signaling on endosomes has been demonstrated by certain so-called class B GPCRs that contain serine/threonine clusters (phosphorylation acceptor sites) in the C-terminal tail [31] that is required for stable β-arrestin binding. Thus, angiotensin II type 1 receptor (AT1R) internalization to endosomes and packaging into exosomes has been shown to be impaired by silencing β-arrestin1 and β-arrestin2 [32]. Therefore, exosomal processing of S1P_2_ *via* endosome trafficking/MVB formation might be defined by the presence of serine/threonine clusters in the C-terminal tail of S1P_2_ and therefore its ability to form stable complexes with β-arrestin. Indeed, S1P_1_, S1P_2_ and S1P_4_ contain such serine/threonine clusters in their C-terminal tails.

The S1P_2_ antagonist, JTE-013 failed to block the CM-stimulated activation of ERK-1/2, suggesting that the S1P_2_ that is taken up and processed by fibroblasts becomes constitutively active. Therefore, the response is not blocked by JTE-013, which functions as a competitive antagonist with S1P for S1P_2_. We have also demonstrated here that a truncated form of S1P_2_ lacking the N-terminal sequence region that contributes to the ligand binding site led to the constitutive activation of ERK-1/2 when expressed in HEK293 cells. Constitutive activation of S1P_2_ might therefore occur as a consequence of proteolysis at the N-terminus, which may normally serve to tension TM1 and (in the absence of bound agonist) stabilise an inactive basal-state conformation to constrain S1P_2_ signaling. Removal of the N-terminal sequence would relieve this inhibitory constraint. The ability of the short constitutively active form of S1P_2_ to stimulate the ERK-1/2 pathway occurs presumably because the receptor adopts a specific conformation that is generated after removal of the N-terminus and that enables formation of active signaling complexes that are competent to regulate the ERK-1/2 pathway.

There are other examples where N-terminal proteolysis of GPCRs can modulate signaling. First, protease activated receptors (PARs) contain their own ligand within their N-terminal regions [33]. This ‘tethered ligand’ becomes accessible and able to activate the receptor when the N-terminus is proteolysed by, for instance, thrombin or trypsin. In addition, the thyroid-stimulating hormone (TSH) receptor is subject to a metalloprotease-mediated cleavage between the membrane-spanning domain and the large N-terminal extracellular domain of the receptor and this leads to activation of the receptor by removal of an inhibitory N-terminal domain, *without* requirement for a tethered ligand. The extracellular domain suppresses constitutive activity of the transmembrane domain of the human TSH receptor [34, 35]. GPCRs can also be inactivated by N-terminal proteolysis e.g. GPR37L1 (an orphan GPCR) [36] and the endothelin ET_B_ receptor [37].

Our findings point to a novel functional signaling role for the processed short form of S1P_2_ that is released from breast cancer cells in its native form in exosomes. The question remains as to whether this short form of the receptor has a role in regulating inter-cellular communication between cancer cells and fibroblasts and whether it can enhance metastatic spread. It is tempting to speculate that the exosomal release of S1P_2_ and its subsequent processing may have a role because exosomal cargoes do play a role in tumour dissemination by influencing and modulating the tumour microenvironment, enabling stromal cells to receive oncogenic and pro-angiogenic signals [38–41]. There are numerous examples of communication between cancer cells and recipient cells involving exosomes in cancer. For instance, exosomes derived from nasopharyngeal carcinoma contain latent membrane protein 1 (LMP1) and are able to induce ERK and AKT activation in the recipient cells, such as epithelial cells, endothelial cells and fibroblasts [42]. In addition, mesenchymal stem cell-derived exosomes regulate MCF-7 breast cancer cell migration involving Wnt signaling [43]. We have shown that the short form of S1P_2_ can promote fibroblast proliferation, although it will be important to establish whether a similar mechanism operates in tumour-associated fibroblasts. The processed short S1P_2_ receptor might function to increase proliferation and therefore the number of fibroblasts that can then be programmed by other factors to undergo transformation to myofibroblasts capable of communicating with cancer cells to promote their dissemination.

Future studies will require characterisation of the signaling properties of the short S1P_2_ receptor and its role in metastatic spread, particularly with regard to tumour-associated fibroblasts. This can be explored by over-expression of the short S1P_2_ form in tumour-associated fibroblasts *in vivo* in order to establish the effect on metastatic spread in breast cancer orthotopic mouse models. In conclusion, our current findings establish a novel role of S1P_2_ in the cancer cell-fibroblast interaction.

## MATERIALS AND METHODS

### Materials

All biochemicals were from Sigma-Aldrich (Poole, UK) unless indicated otherwise. Cell culture media and antibiotics were from Gibco (Paisley, UK) and foetal calf serum from Sera Laboratories International (Haywards Heath, UK). Plasmid constructs for pEGFP hsp70 (# 15215) and pEF6. mCherry-tsg101 (# 38318) were from Addgene (Cambridge, MA, USA) and for HA-tagged S1P_2_ (EDG050TN000) from UMR cDNA Resource Center (Bloomsberg, USA). Lipofectamine 2000™ was from Invitrogen (Leicester, UK), ON-TARGETplus SMARTpool^®^ S1P_2_ siRNA and DharmaFECT 4 from Dharmacon (Cromlington, UK), scrambled siRNA (ALLSTARS Negative control) and endotoxin-free plasmid preparation kits from Qiagen (Manchester, UK). Antibodies employed detected S1P_2_ (PA5-23208) and CD63 (10628D) (ThermoFisher Scientific, UK), S1P_4_ (CAY13489, Cambridge Bioscience Ltd, Cambridge, UK), ERK-2 (610104, BD Biosciences, UK), actin (A2066, Sigma-Aldrich, UK), mCherry (TA150125, OriGene EU, Herford, Germany) and, from Insight Biotechnology Ltd (Wembley, UK), GFP (sc-9996), HA-tag (sc-7392) and phospho-ERK-1/2 (sc-7383). Methyl-[^3^H] thymidine (NET027L) was purchased from Perkin Elmer (Buckinghamshire, UK). CYM5520 and JTE-013 were purchased from Cambridge Biosciences (Cambridge, UK). VECTASHIELD® Hard Set™ Mounting Medium with DAPI was purchased from Vector Laboratories Ltd (Peterborough, UK).

### Cell culture

MDA-MB-231 and MDA-MB-453 breast cancer cells, mouse embryonic fibroblasts (MEFs) and HEK293 cells were maintained in Dulbecco's modified Eagle's media (DMEM) with Glutamax containing 10% (v/v) European foetal calf serum (EFCS) and 1% (v/v), penicillin and streptomycin (Pen-Strep) in humidified conditions at 37 °C in 95% air/5% CO_2_.

### S1P_2_ cloning

N-Terminal 3x HA-tagged full-length human *S1P_2_* in pcDNA3.1(+) was used to create N-terminal 1x and 2x HA-tagged truncated versions of human *S1P_2_* lacking the first 37 amino acids from the predicted amino acid sequence of S1P_2_ via sub-cloning. All of the plasmid DNA used in this study was purified using endotoxin-free plasmid preparation kits and their sequences validated by Sanger sequencing (GATC, Cologne, Germany).

### Transfection with DNA

MDA-MB-231 breast cancer cells were seeded in 75 cm^2^ flasks at 3 × 10^6^ cells/ml complete medium. At approximately 75% confluence, the medium was replaced with Opti-MEM (10 ml) and the cells transfected with 10 μg of DNA plasmid, using Lipofectamine ™ 2000 and incubated for 24 hours. Plasmid constructs encoded either pEGFP hsp70, pEF6. mCherry-tsg101 or N-terminal 3x HA-tagged S1P_2._ HEK293 cells, grown in 12 well plates, were transfected with 1 μg DNA per well using plasmid encoding N-terminal 3x HA-tagged S1P_2_ or N-terminal 1x or 2x HA-tagged truncated S1P_2_ and Lipofectamine ™ 2000 for 24 hours, according to the manufacturer's instructions.

### Transfection with siRNA

MDA-MB-231 cells at approximately 60% confluence were treated with scrambled siRNA or S1P_2_ siRNA oligonucleotide at a final concentration of 100-200 nM in antibiotic-free DMEM and 10% (v/v) EFCS using DharmaFECT 4 and incubated for 24 hours.

### Cell lysate preparation

Cells were lysed in SDS-PAGE sample buffer containing SDS, 0.5% (w/v), Tris, 62.5 mM, pH 6.7, EDTA, 1.25 mM, sodium pyrophosphate, 0.5 mM, DTT, 50 mM, bromophenol blue, 0.06% (w/v) and glycerol, 12.5% (v/v).

### Western blotting

Western blot analysis was performed as described in [44].

### [^3^H] thymidine incorporation and P-ERK-1/2 assay

MEFs were grown to approximately 75-80% confluency on 24 wells plates before quiescing in DMEM for 24 hours. MEFs were then treated with NCM or CM for 10 min and samples taken for western blot analysis with anti-phospho ERK-1/2 antibody. MEFs were also treated with CM for 24 hours and [^3^H] thymidine (9.25 kBq/ml) was added to each well for the final 5 hours. Incubations were terminated by replacing the medium with a brief wash of 1 ml ice cold PBS, followed by the addition of 1 ml ice cold 10% (w/v) TCA for 10 minutes. After 3 × 10 minute washes with TCA (10% w/v), 0.25 ml of 0.1% (w/v) SDS with 0.3 M NaOH was added to each well, the samples transferred to scintillation fluid and the radioactivity incorporated into newly synthesised DNA quantified by scintillation counting.

### Conditioned medium

MDA-MB-231 or MDA-MB-453 breast cancer cells were seeded in 75cm^2^ flask at 3 × 10^6^ cells/ml complete medium and grown to approximately 75% confluence. The adhered cells were gently washed twice with 10 ml of Opti-MEM and then incubated with 10 ml/flask of fresh Opti-MEM for 48 hours before collection of the medium. Cell debris was removed by centrifugation (180*g*, 5 minutes). The resulting supernatant was filtered (0.22 μm, Merck Millipore) to produce conditioned medium (CM). Where indicated, MDA-MB-231 cells were transiently transfected with GFP-hsp70 or mCherry tsg101 or HA-S1P_2_ plasmid construct.

### Protein precipitation

CM was mixed with 125 μl of 6 M TCA per 1 ml of CM and the mixture was shaken well and incubated on ice for a minimum of 2 hours. Following incubation the mixture was transferred into centrifuge tubes (Beckman, Z50901SCA) and centrifuged at 16,125 *g* at 4 °C for 10 minutes using SW40Ti rotor in Beckman-Coulter ultracentrifuge XL-100K. The supernatant was discarded and pellets (PPT) re-suspended in 40 μl of 9 M urea in 50 mM Tris (pH 7.5). SDS-PAGE sample buffer was added and 2M Tris to neutralise. Samples were stored at -20 °C for future western blot analysis.

### Isolation of exosomes

Conditioned media from a minimum of three 75cm^2^ flasks of MDA-MB-231 cells was centrifuged at 20,000 *g* (4^°^C, 30 minutes) and the resulting supernatant ultra-centrifuged at 100,000 *g* at 4 °C for 75 minutes. The exosome pellet was re-suspended in 1 ml of sterile filtered phosphate buffered saline (PBS) pH 7.4 at 4 °C and the ultra-centrifugation step repeated to further purify the exosomes. The final exosome pellet was re-suspended in 250μl sterile filtered PBS, pH 7.4 and stored at -80°C.

### Immunofluorescence microscopy

MEFs or MDA-MB-231 cells were seeded at density of 50 × 10^4^ cells/ml complete medium on 13 mm cover slips in a 12 well plate. MEFs were quiesced for 24 hr before treatment with either non-conditioned medium (NCM + Opti-MEM) or CM or CYM5520. Cells on coverslips were washed with cold PBS pH 7.4, fixed with 3.7% (v/v) formaldehyde in PBS pH 7.4 at 4 °C for 10 minutes and permeabilized with 0.1% (v/v) Triton X-100 in PBS pH 7.4 (1ml/well) for 2 minutes. After two washes with cold PBS pH 7.4, non-specific binding of antibodies was blocked using blocking solution containing 5% (v/v) EFCS, 10 mg/ml BSA in PBS pH 7.4 for 30 minutes. Proteins of interest were detected using specific antibodies (diluted 1:100 with blocking solution), incubated for 1 hour at room temperature. After washing three times with ice cold PBS pH 7.4, secondary antibodies conjugated to either TRITC or FITC or Texas Red (diluted 1:100 with blocking solution) were used to detect proteins of interest. Coverslips were mounted using VECTASHIELD® Hard Set™ Mounting Medium with DAPI. Samples were stored at 4 °C or directly examined for immunofluorescence by confocal microscopy (Leica TCS SP5).

### Electron microscopy detection of exosome proteins using immuno-gold labelling

Exosomes were re-suspended in an equal volume of 4% paraformaldehyde/PBS and 5 μl droplets of placed onto poly-L-lysine/carbon coated nickel grids. Grids were washed (6 × 1 minute) with PBS to remove paraformaldehyde followed by 0.05 M Glycine/PBS (3 × 5 minutes) before blocking with 3% BSA/PBS (3 × 5 minutes) and then adding anti-CD63 antibody (1:100), anti-S1P_2_ antibody (1:100) or no antibody (blocking solution only). After 1 hour, the grids were washed with 3% BSA/PBS (6 × 5 minutes) before incubation (1 hour) with gold-labelled secondary antibodies. Gold (15 nm)-labelled goat anti-mouse IgG (1:20) was used to detect CD63; gold (10 nm)-labelled anti-rabbit IgG (1:20) to detect S1P_2_. No antibody controls were processed with both gold-labelled secondary antibodies. After further washes with blocking buffer (6 × 5 minutes) and PBS (6 × 5 minutes), grids were prepared for visualisation and imaged using a Gatan Multiscan 794 camera and a 200 kV FEG high resolution FEI Tecnai T20 transmission electron microscope

### Densitometirc and statistical analysis

Densitometric values quantified using ImageJ were expressed as the ratio of P-ERK/actin (represented as mean +/− SEM for fold changes, n=3 experiments). Statistical analysis was undertaken using one way ANOVA or t-test and considered significant when ^*^p < 0.05.

## SUPPLEMENTARY MATERIALS AND FIGURES



## Abbreviations

CM: conditioned medium
CYM5520: 1-[2-[2,5-dimethyl-1-(phenylmethyl)-1*H*-pyrrol-3-yl]-2-oxoethyl]-1,6-dihydro-6-oxo-3-pyridinecarbonitrile
EMT: epithelial-mesenchymal transition
ERK-1/2: extracellular signal regulated kinase 1/2
EV: extracellular vesicles
GPCR: G protein-coupled receptor
hsp70: heat shock protein 70
JTE-013: 1-[1,3-dimethyl-4- (2-methylethyl)-1*H*-pyrazolo[3,4-*b*]pyridin-6-yl]-4-(3,5- dichloro-4-pyridinyl)-semicarbazide;
MEF: mouse embryonic fibroblasts
MVB: multi-vesicular bodies
NCM: non-conditioned medium
S1P_2_: sphingosine 1-phosphate receptor 2
S1P: sphingosine 1-phosphate
SK1: sphingosine kinase 1
SK2: sphingosine kinase 2
TM: transmembrane domain
tsg101: tumor susceptibility gene 101.
