# Supplementary material for: The sphingosine 1-phosphate receptor 2 is shed in exosomes from breast cancer cells and is N-terminally processed to a short constitutively active form that promotes extracellular signal regulated kinase activation and DNA synthesis in fibroblasts
Source: Oncotarget. 2018 Jun 29;9(50):29453–67. doi: 10.18632/oncotarget.25658 (PMC6047680; doi:10.18632/oncotarget.25658)
Supplement: Supplementary file 1 [file oncotarget-09-29453-s001.pdf]

The sphingosine 1-phosphate receptor 2 is shed in exosomes from breast cancer cells and is N-terminally processed to a short constitutively active form that promotes extracellular signal regulated kinase activation and DNA synthesis in fibroblasts

SUPPLEMENTARY MATERIALS

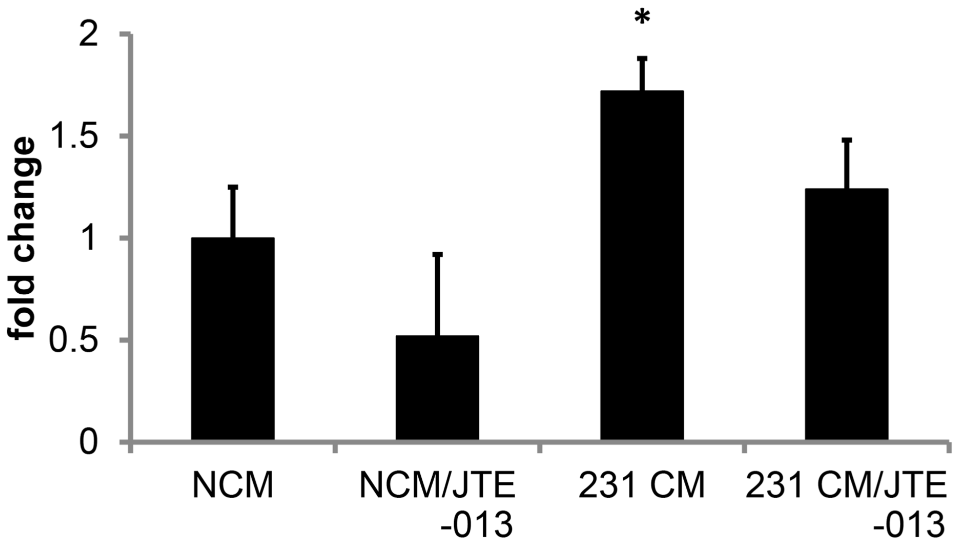

Supplementary Figure 1: Effect of CM from MDA-MB-231 cells on phosphorylated ERK-1/2 levels in MEFs. Bar graph showing the effect of NCM and CM on ERK-1/2 phosphorylation in MEFs treated with vehicle (DMSO, 0.1% v/v) or JTE-013 (10 µM). Data are expressed as the fold change in the P-ERK-1/2: actin ratio and are means +/- SD for n=3 experiments. * p values < 0.05 for CM *versus* NCM.

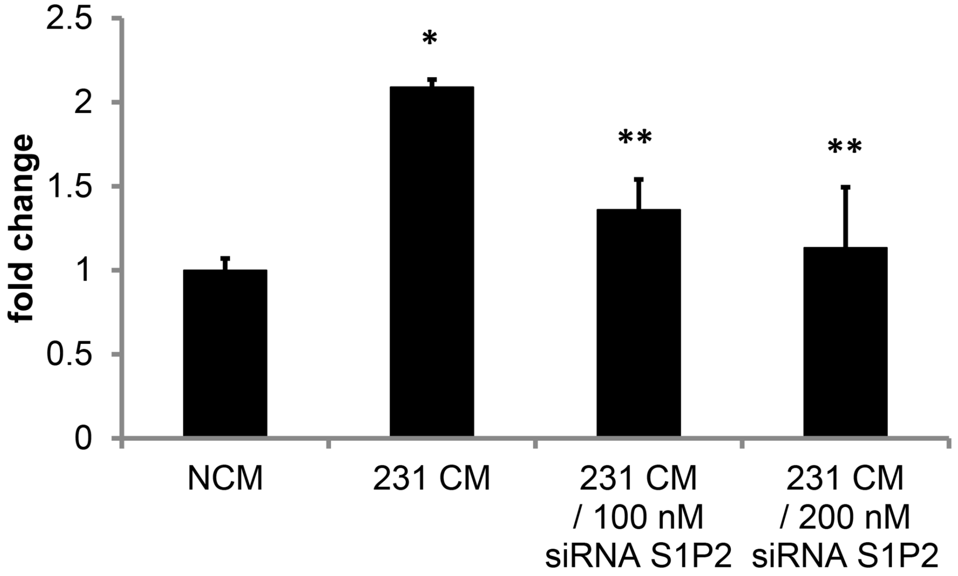

Supplementary Figure 2: Specificity for ERK-1/2 activation in MEFs for $S1P_2$ in CM from MDA-MB-231 cells. Effect of CM isolated from MDA-MB-231 in which $S1P_2$ is knocked down with siRNA (100 and 200 nM) on phosphorylated ERK-1/2 levels in MEFs. Data are expressed as the fold change in the P-ERK-1/2: actin ratio and are means +/- SD for n=3 experiments. * p values < 0.05 for CM versus NCM and ** p < 0.05 for siRNA/CM *versus* scrambled/CM.

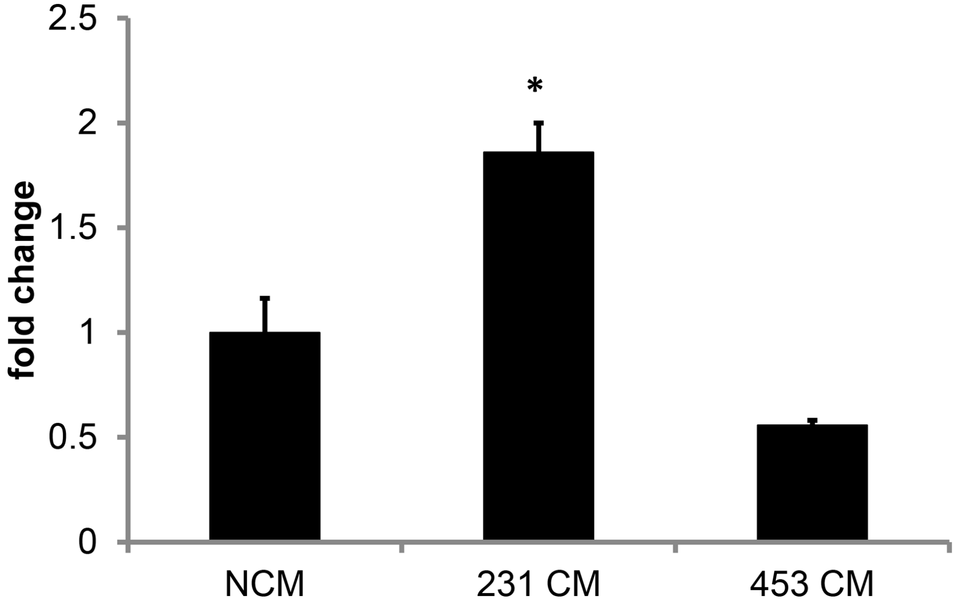

Supplementary Figure 3: Effect of CM from MDA-MB-231 and MDA-MB-453 cells on phosphorylated ERK-1/2 levels in MEFs. Bar graph showing the effect of CM containing either $S1P_2$ or $S1P_4$ from MDA-MD-231 and MDA-MB-453 cells respectively on ERK-1/2 phosphorylation in MEFs. Data are expressed as the fold change in the P-ERK-1/2: actin ratio and are means +/- SD for n=3 experiments. * p values < 0.05 for 231 CM *versus* NCM.

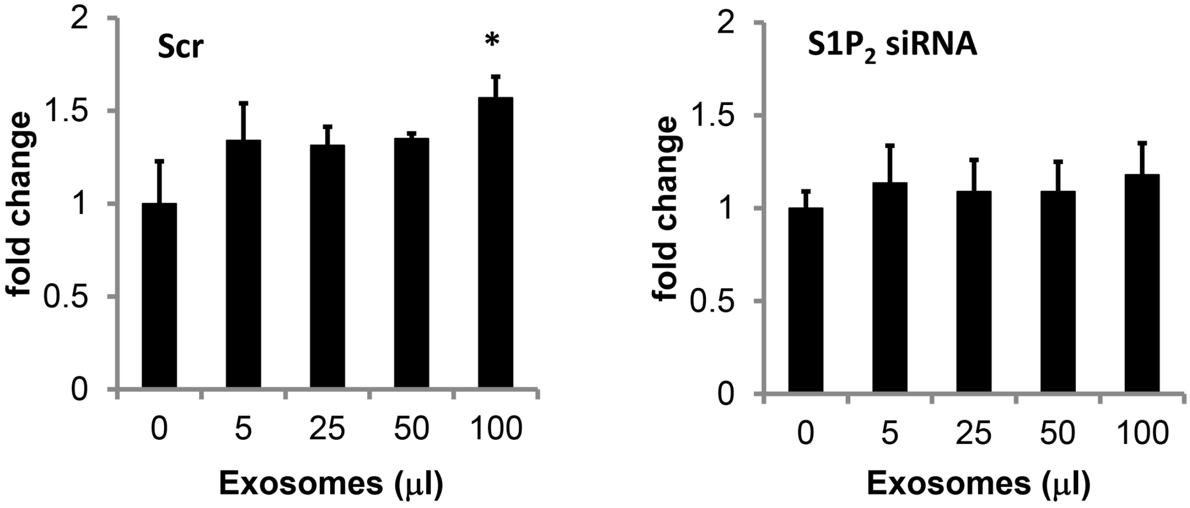

Supplementary Figure 4: Effect of exosomes containing $S1P_2$ from MDA-MB-231 on phosphorylated ERK-1/2 levels in MEFs. Bar graph showing the effect of exosomes isolated from scrambled or $S1P_2$ siRNA-treated MDA-MB 231 cells on ERK-1/2 phosphorylation in MEFs. Data are expressed as the fold change in the P-ERK-1/2: actin ratio and are means +/- SD for n=3 experiments. * p values < 0.05 for Exo *versus* PBS.
